# Bile acids and the gut microbiome are involved in the hyperthermia mediated by 3,4-methylenedioxymethamphetamine (MDMA)

**DOI:** 10.1038/s41598-024-65433-2

**Published:** 2024-06-24

**Authors:** Srishti Rana, Jeremy R. Canfield, Christopher S. Ward, Jon E. Sprague

**Affiliations:** 1 Department of Biological Sciences, Bowling Green State University, Bowling Green, OH 43403 USA; 2 The Ohio Attorney General’s Center for the Future of Forensic Science, Bowling Green State University, Bowling Green, OH 43403 USA

**Keywords:** Toxicology, Pharmacodynamics

## Abstract

Hyperthermia induced by phenethylamines, such as 3,4–methylenedioxymethamphetamine (MDMA), can lead to life-threatening complications and death. Activation of the sympathetic nervous system and subsequent release of norepinephrine and activation of uncoupling proteins have been demonstrated to be the key mediators of phenethylamine-induced hyperthermia (PIH). Recently, the gut microbiome was shown to also play a contributing role in PIH. Here, the hypothesis that bile acids (BAs) produced by the gut microbiome are essential to PIH was tested. Changes in the serum concentrations of unconjugated primary BAs cholic acid (CA) and chenodeoxycholic acid (CDCA) and secondary BA deoxycholic acid (DCA) were measured following MDMA (20 mg/kg, sc) treatment in antibiotic treated and control rats. MDMA-induced a significant hyperthermic response and reduced the serum concentrations of three BAs 60 min post-treatment. Pretreatment with antibiotics (vancomycin, bacitracin and neomycin) in the drinking water for five days resulted in the depletion of BAs and a hypothermic response to MDMA. Gut bacterial communities in the antibiotic-treated group were distinct from the MDMA or saline treatment groups, with decreased microbiome diversity and alteration in taxa. Metagenomic functions inferred using the bioinformatic tool PICRUSt2 on 16S rRNA gene sequences indicated that bacterial genes associated to BA metabolism are less abundant in the antibiotic-MDMA treated group. Overall, these findings suggest that gut bacterial produced BAs might play an important role in MDMA-induced hyperthermia.

## Introduction

The use of phenethylamine psychostimulants, such as 3,4–methylenedioxymethamphetamine (MDMA) have been associated with hyperthermia and hyperthermic sequelae, including disseminated intravascular coagulation, rhabdomyolysis, kidney failure and even death^1^. During phenethylamine-induced hyperthermia (PIH), the activated sympathetic nervous system (SNS) and subsequent increase in plasma norepinephrine (NE) levels^2^ have been established as the primary mediators of PIH^1^. NE directly or indirectly promotes lipolysis in fat reservoirs such as brown and white adipose tissues (BAT and WAT) and releases free fatty acids (FFA) within BAT or into systemic circulation from WAT^2^. These FFAs subsequently activate mitochondrial uncoupling proteins (UCPs) to generate heat, where UCP1 and UCP3 play complementary roles in the onset and maintenance of PIH, respectively^3^. Additionally, NE also prevents heat dissipation through α_1_-adrenergic receptor mediated vasoconstriction^4^. Recently, the roles of non-host contributors, particularly the gut microbiome, have been recognized for their influence on PIH^5,6^. Application of gut bacterial modulation methods such as antibiotic treatment^5^ and fecal microbial transplantation (FMT)^6^ showed that gut bacteria are likely playing a contributing role in PIH. However, the specific mechanism(s) by which the gut microbiome could affect PIH has not been elucidated.

Bile acids (BAs) are 24-carbon steroids produced by the mammalian liver for digestion of dietary fats^7^, some portion of which undergo various bacterial transformations (e.g., deconjugation, 7α-dehydroxylation) in the gut to produce different species of BAs^8^. Alteration in gut bacterial compositions and/or metabolites/products levels, including different species of BAs, have been observed and related to various forms of thermogenesis. One example of this has been shown during adaptive thermogenesis induced by cold exposure in the presence or absence of high fat diet (HFD)^9–12^ and through fat burning in HFD induced obesity^13–15^. Zietak et al.^10^ reviewed the link between intestinal bacteria, BAs and UCP regulated thermogenesis. BAs can increase energy expenditure in a UCP-dependent fashion in BAT and skeletal muscle (SKM)^13^, where they activate the G-protein coupled bile acid receptor, Takeda G protein receptor 5 (TGR5)^16^. Based upon these observations, we hypothesized that gut microbiome-produced BAs may be contributing to PIH.

**Figure 1:**
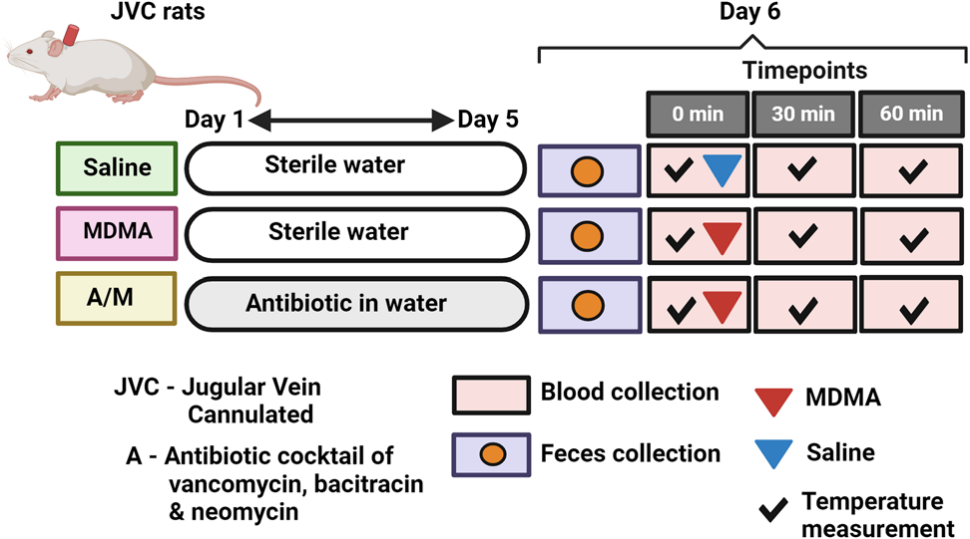
Schematic of the study’s experiment design. JVC rats were randomly divided into Saline, MDMA and A/M groups (n = 5 or 6), where groups MDMA and Saline had access to sterilized water while A/M was provided with antibiotic mixed water for 5 days. On Day 6, fecal pellets were collected right before the MDMA/Saline challenge from rats in all three groups that were individually caged in a sterilized bedding for past 24 h. This was followed by temperature measurements and blood draws at three timepoints: 0 min, 30 min and 60 min., where 0 min represents timepoint right before MDMA (20 mg/kg, sc.) or Saline administration and 30 min and 60 min post MDMA/Saline treatment. Fecal and blood samples (serum) were stored at − 80 °C and − 20 °C until analysis. For microbial analysis, all three groups: MDMA, A/M and Saline represent feces collected before the MDMA/Saline challenge. Figure made in BioRender.

In the present study, we measured changes in serum concentrations of unconjugated primary BAs cholic acid (CA) and chenodeoxycholic acid (CDCA) and secondary BA deoxycholic acid (DCA) following a hyperthermic dose of MDMA in antibiotic treated and control rats (Fig. 1). We further inferred functional potential of the same animals’ gut microbiomes using PICRUSt2^17^ to examine predicted abundance changes in the major bacterial genes that encode BA-metabolizing enzymes.

## Results

### MDMA-induced change in core body temperature

**Figure 2:**
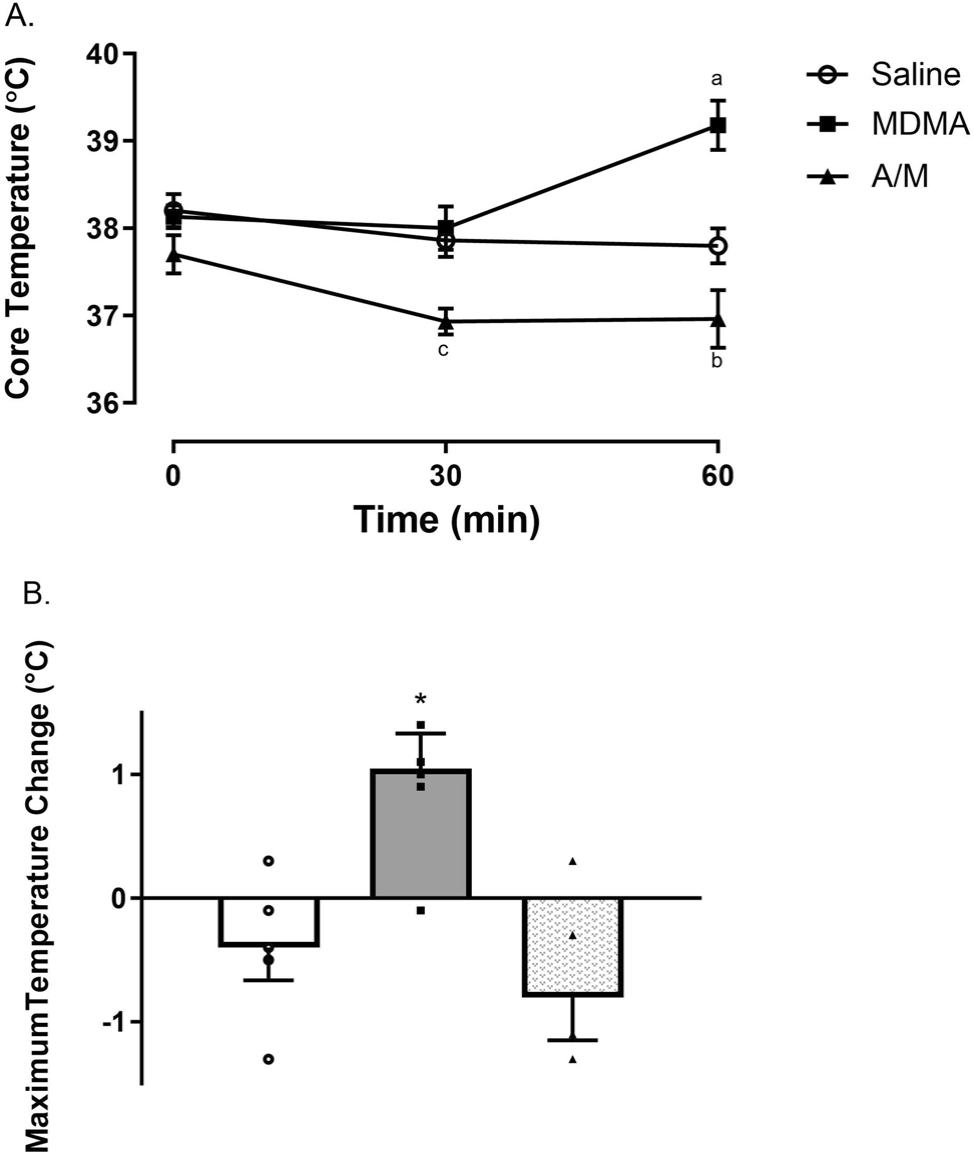
(**A**) Core temperature measurements for animals in all three treatment groups (saline, MDMA and A/M) at three different time-points (0, 30 and 60 min). (**B**) Maximum temperature change (°C) observed for the same groups over the three time-points. Each value represents mean ± SEM, n = 5 or 6. ^a^denotes a significant difference between saline and MDMA group (One-way ANOVA F_(2,6)_ = 5.364; p = 0.04 with a Student Newman-Keuls post hoc test, p < 0.01), ^b^indicates MDMA group being significantly different from A/M group (One-way ANOVA F_(2,6)_ = 5.364; p = 0.04 with a Student Newman-Keuls post hoc test, p < 0.001), ^c^indicates significant temperature difference between the saline and the A/M group, as well as MDMA and A/M group (One-way ANOVA F_(2,6)_ = 5.364; p = 0.04 with a Student Newman-Keuls post hoc test, p < 0.01). *Maximum change in temperature was significant between MDMA and saline group, also between MDMA and A/M group (F_(2,13)_ = 11.096; p < 0.0015) using a one-way ANOVA with Student–Newman–Keuls with post-hoc test.

MDMA-mediated an increase in core body temperature that was significantly different compared to both the saline (One-way ANOVA F_(2,6)_ = 5.364; p = 0.04) and the antibiotics** + **MDMA (A/M) (One-way ANOVA F_(2,6)_ = 5.364; p = 0.04) treatment groups at the 60 min time point (Fig. 2A). The temperatures of animals in the saline treatment group were steady (ranging from an average of 37.8 °C to 38.2 °C) across all three time points with no significant differences noted. The A/M group demonstrated a hypothermic response following MDMA treatment. Analysis of the maximum temperature change shows that MDMA resulted in a significant maximal temperature change of + 1.05 °C (One-way ANOVA with Student–Newman–Keuls with post-hoc test shows the A/M group displayed a significant decrease (F_(2,13)_ = 11.096; p < 0.0015; Fig. 2B).

### Unconjugated serum BA levels decrease during MDMA induced hyperthermia

**Figure 3:**
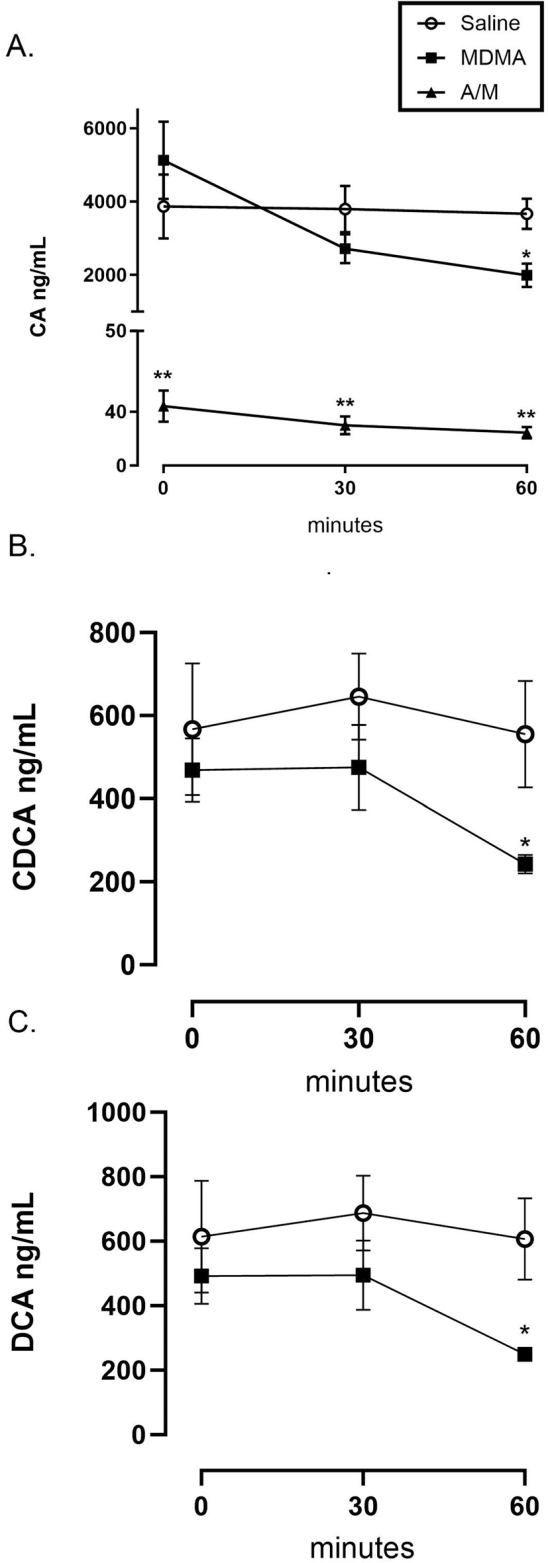
Serum bile acid levels at three different time points, pre (0 min) and post (30 min and 60 min) MDMA (20 mg/kg, sc.) or Saline administration in antibiotic treated or untreated male Sprague–Dawley rats. (**A**) Change in Cholic Acid (CA) levels; (**B**) Change in Chenodeoxycholic Acid (CDCA) levels and (**C**) Change in Deoxycholic Acid (DCA) levels. Both CDCA and DCA levels were below detection limits of 5 ng/mL in the A/M treatment group, hence not shown in the Figures. Each value is the mean ± SEM (n = 5 or 6). * Indicates significant difference between MDMA and saline/control value at the same time point p < 0.05 using a t-test. ** Indicates significant difference between A/M and all other treatment groups (F_(2, 9)_ = 25.266; one-way ANOVA with Student–Newman–Keuls post hoc test; p < 0.0002).

Of the three serum BA species measured, CA was found to be the most abundant BA in the saline and MDMA treatment groups with mean baseline concentrations of 3866.64 ± 870.22 ng/mL and 5124.44 ± 1052.30 ng/mL respectively. In these same treatment groups, the mean baseline concentration for CDCA and DCA were also found to be in a similar range: 567.18 ± 158.93 ng/mL and 468.20 ± 76.88 ng/mL for CDCA and 614.00 ± 173.19 ng/mL and 492.39 ± 86.53 ng/mL for DCA. Antibiotic treatment before MDMA administration lowered the CA levels to 44.23 ± 11.69 ng/mL and CDCA and DCA levels were below the detection limit of 5 ng/mL (Fig. 3A).

Sixty minutes following MDMA treatment, CA levels in the MDMA group decreased by 45.67% to 1991.07 ± 316.79 ng/mL which was significantly lower compared to the saline (F_(2, 9)_ = 25.266; one-way ANOVA with Student–Newman–Keuls post hoc test; p < 0.0002) at the same time point and the basal (F_(2,8)_ = 11.145; Repeated measures ANOVA with Dunnett post-test; p < 0.0049) levels within the MDMA group (Fig. 2A). A t-test revealed that the CDCA levels (Fig. 3B) were significantly reduced by 56.43% to a mean value of 242.62 ± 22.02 ng/mL at 60 min compared to saline (t-test; p < 0.05) and basal levels (Repeated measures ANOVA’s Dunnett F_(2,8)_ = 8.325, p < 0.05 for 0 vs 60 min) within the MDMA treatment group. DCA followed the same pattern displaying a significant reduction of 58.86% to 249.84 ± 20.75 ng/mL at 60 min compared to saline (t-test; p < 0.008) at that time point and basal levels (Repeated measures ANOVA’s Dunnett F_(2,8)_ = 7.968, p < 0.05 for 0 vs 60 min) within the MDMA treatment group (Fig. 3C).

### Antibiotic treatment results in depletion of the microbial diversity

**Figure 4:**
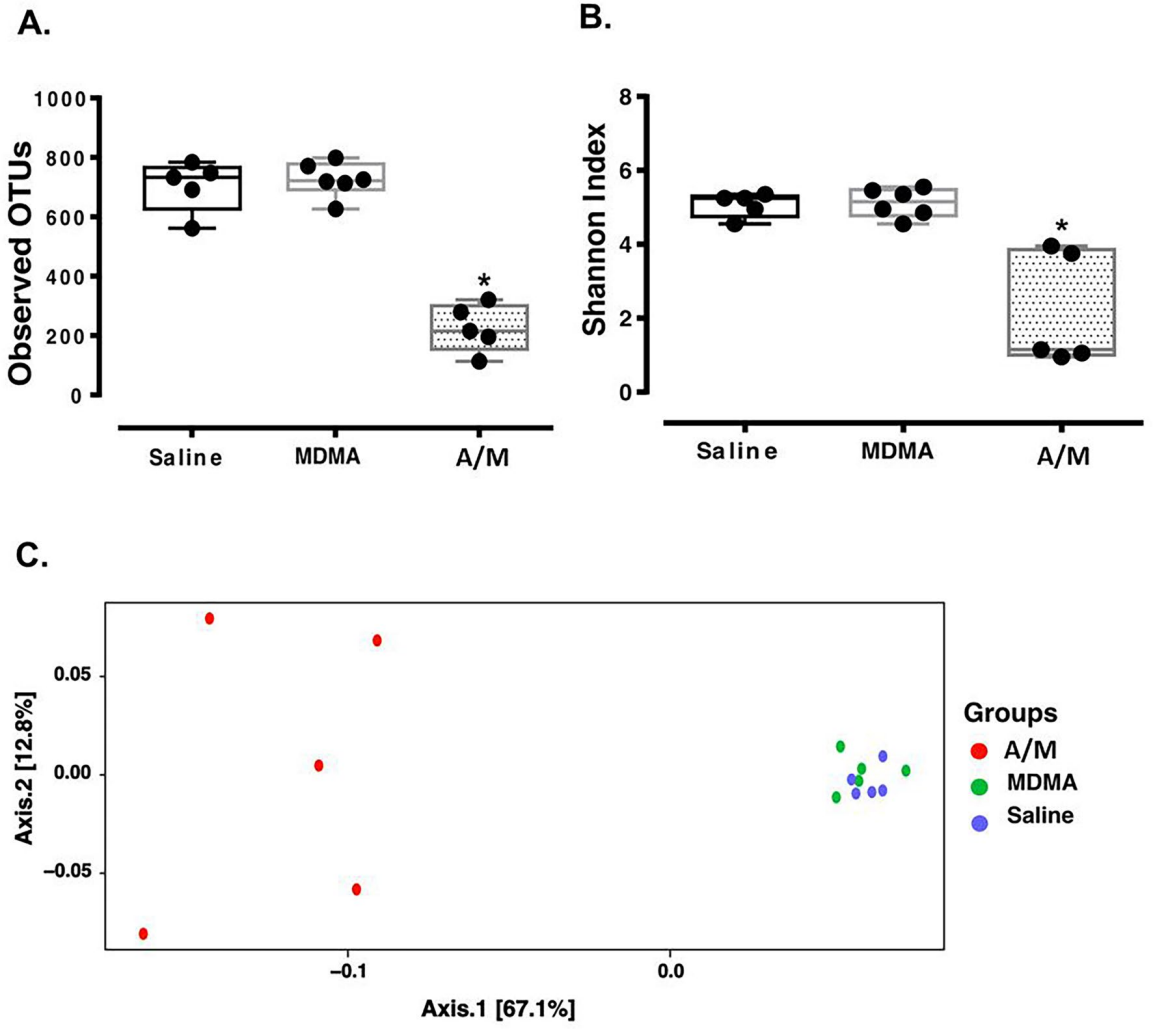
Alpha and beta diversities. Panel (**A**) Observed ASVs and Panel (**B**) Shannon Index values for the three treatment groups. All points are shown in box and whisker plots with line representing median values for all groups. *denotes difference between Saline and A/M as well as MDMA and A/M with (Kruskal–Wallis pairwise post-hoc Dunn test with both saline and MDMA, p < 0.05). Panel (**C**) PCoA plot based on weighted Unifrac method showing significant difference between A/M, saline and MDMA groups (p < 0.013, R^2^ = 0.38; PERMANOVA). MDMA, A/M and Saline represent feces collected before the MDMA/Saline challenge.

Analysis of gut bacterial communities from fecal samples collected after 5 days of sterile drinking water or antibiotic laced water showed that antibiotic treatment effectively altered the microbiome compositions (Fig. 4). While median ASV richness (OTUs observed) were 722 and 733 for the saline and MDMA groups, the A/M group had a comparatively lower median ASV richness of 215 (Kruskal–Wallis pairwise post-hoc Dunn test with saline and MDMA, p < 0.05; Fig. 4A). Similarly, median Shannon Diversity values were 5.3 and 5.2 for the saline and MDMA groups, whereas the median Shannon Diversity value in the antibiotic group was 1.1 (Kruskal–Wallis pairwise post-hoc Dunn test with saline and MDMA, p < 0.05; Fig. 4B). Antibiotic treatment also induced changes in community composition (Fig. 4C). Differences between groups (i.e., beta diversity) were significant (p < 0.013, R^2^ = 0.38; PERMANOVA).

**Figure 5:**
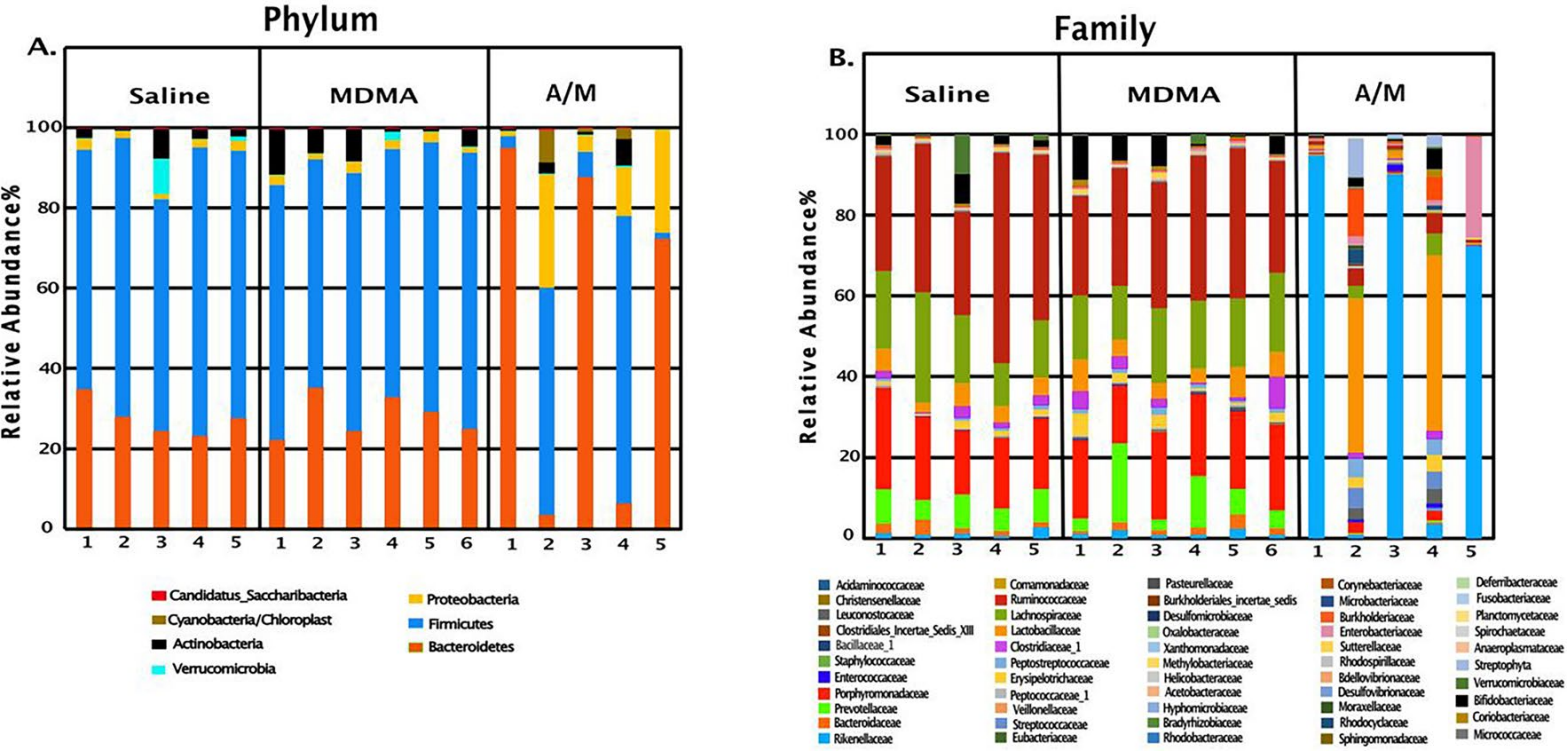
Figure showing relative abundance of major phyla (**A**) and families (**B**) for three treatment groups. Numbers on X-axis represent individual animals in each group. MDMA, A/M and Saline represent fecal samples collected before the MDMA/Saline challenge.

Saline and MDMA groups had similar taxonomic profiles, while the A/M group showed distinctly altered taxonomic profiles (Fig. 5). Firmicutes, Bacteroidetes, Actinobacteria, Proteobacteria and Verrucomicrobia were the most abundant phyla, in the order of their abundance in the MDMA and saline groups (Fig. 5A). Within the A/M group, there were two divergent profiles: one half of the samples comprised predominantly Bacteroidetes (> 72%) and depleted Firmicutes (6.2% or less). Conversely, the other half displayed the opposite trend with depleted Bacteroidetes (6.4% or less) and predominantly Firmicutes (56.5% or more). The antibiotic treatment group also had elevated levels of Proteobacteria (yellow bars, Fig. 5A). Antibiotic treatment also eliminated Lachnospiraceae and Ruminococcaceae from Firmicutes phylum and Prevotellaceae and Porphyromonadaceae from Bacteroidetes (Fig. 5B) group. Rikenellaceae (Bacteroidetes), Lactobacillaceae (Firmicutes) and Burkholderiaceae (Proteobacteria) were the dominant families present in the antibiotic treated group.

### Bacterial BA gene abundances are altered in antibiotic-depleted microbiomes

PICRUSt2 was used to infer the functional genes (metagenome) of bacteria identified from 16S rRNA genes in the sequenced microbial communities. The nearest sequenced taxon index (NSTI) values that indicate the extent of likeliness of bacteria (16S rRNA gene) being similar to or represented in the reference database, for all samples in this experiment was found between 0.02 and 0.13 (lower values for antibiotic group), with a mean weighted NSTI value of 0.08 ± 0.01. These lower values suggest that the observed ASVs or their very close relatives were highly represented in the reference database applied by PICRUSt2, which results in much closer predictions of a community’s functional gene content^18^.

We specifically focused on the relative abundances (genes per total metagenome) of inferred bacterial genes coding for enzymes involved in BA metabolism, which are bile salt hydrolase (BSH, EC:3.5.1.24), *bai* (bile acid inducible) genes in the *bai* operon. We tested MetaCyc, which is a curated database as the major reference database and additionally Kyoto Encyclopedia of Genes and Genomes (KEGG) for their performance in inferring gene content.

**Figure 6:**
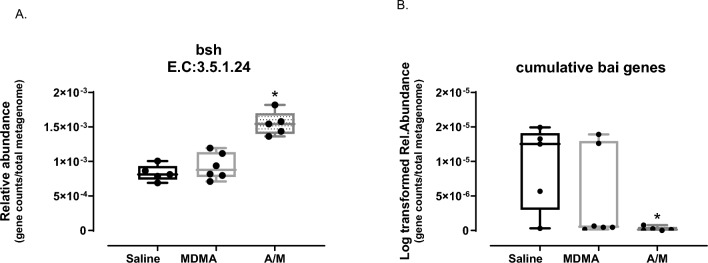
PICRUSt2 based relative gene abundance (normalized per total genes in predicted metagenome) from bacteria associated to bile acid metabolism. (**A**) Bile Salt Hydrolase (BSH) or Choloylglycine hydrolase gene was significantly different between Saline and A/M as well as MDMA and A/M group (Kruskal–Wallis pairwise post-hoc Dunn test with saline and MDMA, *p < 0.05). (**B**) Log-transformed cumulative *bai* genes, statistically different between Saline and A/M groups (Kruskal–Wallis pairwise post-hoc Dunn test with saline, *p < 0.05). All points are shown in box and whisker plots with line representing median values for all groups. E.C (Enzyme Commission number). BSH genes abundance is based on MetaCyc database and *bai* genes based on KEGG. MDMA, A/M and Saline represent feces collected before the MDMA/Saline challenge.

Genes encoding bile salt hydrolase (BSH, EC:3.5.1.24) enzymes responsible for the first step of bacterial BA metabolism^8^ were found to have median relative abundance of 0.00154 using MetaCyc values within the A/M treated group, which was significantly higher (Kruskal–Wallis pairwise post-hoc Dunn test with both saline and MDMA, p < 0.05) than in the other two groups (median relative abundances of 0.00087 and 0.00081 in the MDMA and saline groups, respectively; Fig. 6A). In the A/M treated group, BSH gene counts were primarily attributed to either the genera *Alistipes* (relative abundance > 72.3%) or *Lactobacillus* (relative abundance > 43.6%), while the distribution of this gene was even across numerous taxa in the other two groups.

The bile acid inducible (*bai*) operon can consist of 8–12 genes that code for enzymes involved in the secondary BA production pathway via 7-alpha-dehydroxylation, which follows the deconjugation step^19^. Of the two databases utilized, MetaCyc did not contain this BA metabolism pathway whereas the KEGG showed elevated secondary BA metabolism in the A/M group. Because microbiome related to secondary BA metabolism is being newly explored in mammalian systems^19^ and therefore may not have been adequately understood or represented in metabolic databases, we attempted MetaCyc and KEGG for inferring the individual *bai* genes content instead. MetaCyc detected only *bai*E and *bai*F genes, whereas KEGG detected a total of 7 genes in the *bai* operon including *bai*A, *bai*B, *bai*CD, *bai*E, *bai*F, *bai*H and *bai*I, whereas MetaCyc detected only *bai*E and *bai*F in saline and MDMA groups. Because KEGG had more BA reference genes for the *bai* operon, we chose to use KEGG to compare bai gene abundance values between groups. Interestingly, *bai* genes were completely undetected in two animals while only *bai*CD and/or *bai*H or *bai*B gene were detected in very low abundance in the rest of the antibiotic group. The other two groups overall had greater presence of *bai* genes. However, in the non-antibiotic groups (particularly within the MDMA group), the *bai* genes were also found in lower abundance in some of the animal samples. For example, *bai*CD and *bai*H were present in all animals, while the remaining *bai* genes were inconsistently present across the treatment groups. Thus, to compare across treatment groups, we calculated the cumulative abundance of all *bai* genes. A/M treated animals had significantly lower *bai* gene abundances than the saline (Kruskal–Wallis pairwise post-hoc Dunn test with saline, p < 0.05) and lower values than MDMA treatment groups (non-significance; median log-transformed values were 1.25 × 10^–5^, 5.45 × 10^–7^ and 1.24 × 10^–7^ respectively for saline, MDMA and A**/**M groups; Fig. 6B). Average log-transformed values were 9.34 × 10^–6^, 3.12 × 10^–6^ and 2.24 × 10^–7^ for the same groups respectively.

## Discussion

In the present study, antibiotics (vancomycin, neomycin and bacitracin) added to the drinking water for five days significantly altered the gut microbiome and reduced the serum levels of unconjugated BAs CA, CDA and CDCA before MDMA treatment. Following MDMA treatment, the animals exposed to the antibiotics displayed a reversal (hypothermia) compared to the MDMA-induced hyperthermia. A number of studies have shown that antibiotics can reduce the hyperthermia mediated by phenethylamines^5,20,21^. Ridge et al. first suggested a potential role for BA in PIH by showing that antagonism of the TGR5 BA receptor with triamterene or deiodinase II (D2) enzyme with iopanoic acid reduced MDMA-mediated hyperthermia^5^.

Human studies have examined changes in circulating levels of various biochemicals associated to post phenethylamine drug use^22–24^. Plasma samples from human subjects post amphetamines and mephedrone for example displayed decreases in BAs, while no change in BA levels was observed post MDMA treatment^24^. These studies predominantly implemented non-targeted LC–MS/MS methods to assess changes in analytes which could limit the levels of detection^24,25^. Zhang et al.^26^ binge treated mice with methamphetamine (15 mg/kg iv. Twice daily) for four consecutive days which ultimately resulted in an increase in total serum BA levels on the fifth day. However, individual BA species were not measured. Those authors did find that fecal unconjugated BA levels but not the conjugated BAs were depleted following methamphetamine treatment. These findings support that (repeated) phenethylamine use, such as methamphetamine and MDMA could affect unconjugated BA levels in the host.

Some studies suggest that secondary BAs such as DCA could be more potent ligands for TGR5 both in vitro and in vivo^14,27–30^ and thus may have stronger signaling properties for host thermogenesis^14,29^. The similar decreasing patterns for all three BA species in the present study, both primary (CA and CDCA) and secondary (DCA) in the MDMA treatment group only suggests the potential involvement of all three unconjugated BAs in PIH. This is further supported by the observation that the decrease in BA occurred at the same time point as the peak MDMA-induced hyperthermic response at the 60 min time point.

We for the first time studied the potential importance of bacterial BA metabolism on PIH via analyses of various major bacterial BA genes in antibiotic treated and untreated animals. We inferred functional gene content of microbiomes from 16S rRNA gene sequences using PICRUSt2. BSH genes were found to have higher relative abundance within the antibiotic group. Microbiome analysis confirms that our antibiotic cocktail was effective in decreasing community diversity and changed the normal microbiome profile, where the total DNA yields from fecal extractions suggested reduced microbial biomass in the gastrointestinal track of antibiotic treated animals. The serum CA and CDCA levels in the antibiotic treated animals is however inconsistent with this abundance in the BSH gene. To confirm this observation, absolute quantitation of BA genes via quantitative PCR is needed.

We observed few dominant ASVs (relative abundance between ~ 70% and 90%) from *Lactobacillus* or *Alistipes* genera (not shown) from Lactobacillaceae (phylum Firmicutes) or Rikenellaceae (phylum Bacteroidetes) families respectively as the main source of BSH genes in the antibiotic treated group. These two genera have previously been identified as important BSH contributors in mammalian gut bacterial community^31,32^, where multiple homologs of BSH genes and/or variability in their phenotype/specificity have been reported in Lactobacillus genus^33^. BSH genes in the groups without antibiotic treatment showed contribution from a large variety of different taxa (without any major contributors), as observed during in silico studies^34^.

The depletion of genes in the *bai* operon that encode enzymes for secondary BA production in the A/M group was consistent with the measured BA profiles. This suggests that the antibiotic combination used in this experiment may be more effective against secondary BA producing bacteria compared to BSH gene harboring bacteria. Vrieze et al. also found decreased levels of secondary BAs and bacteria involved in the production of secondary BAs post vancomycin treatment^35^. However, these genes represent low abundant species in gut bacterial community^36^, for which read counts were not evenly dispersed across all seven *bai* genes as noted in the MDMA only treatment group. These findings suggest the potential for unequal sequencing depth (efficiency) for the relevant bacteria possessing these genes and/or lack of robust databases and annotation. There is, therefore, a need for studies to update the metagenome-inferring tools^37^ or shotgun metagenomic analysis where not all genes in the *bai* operon were detected^36^.

Primary BAs are produced in the liver in an unconjugated or free form, which are then conjugated with taurine or glycine before being released into the gut^38^. Hence, it is to be noted that certain portions of CA and CDCA, the dominant unconjugated primary BA species in the normal mammalian systemic circulation^36,39^, could also come directly from the host^40^. But it is apparently unclear as to what proportion of these circulating BA species undergo bacterial deconjugation^40^. As much of these unconjugated BAs, e.g. CA have been found at diminished levels in serum of germ-free rodents^39^, deconjugation seems to be important for bacterial action on influencing the level of (unconjugated) BA species in host BA pool and hence PIH.

Overall, these findings suggest that the BAs from the gut microbiome play a contributing role to the hyperthermia mediated by MDMA.

## Methods

### Animals

Jugular Vein Cannulated (JVC) male Sprague Dawley (*Rattus norvegicus domesticus*) rats weighing 258.9 ± 18.0 g (7 to 8 weeks old) were obtained from Envigo (Indianapolis, IN). Animals were randomly divided into three groups: Saline, MDMA and antibiotic + MDMA (A/M) and housed one per sterilized cage (cage size: 21.0 × 41.9 × 20.3 cm) in a room maintained on a 12:12 h light/dark schedule and at an ambient temperature of 26–27 °C to maximize thermogenic response^41^. Animals were given a five day acclimation period during which they were provided access to either sterile drinking water (saline and MDMA treatment groups) or antibiotics (A/M) in the sterile drinking water. Animals had ad libitum access to food (10% fat)^42^ and sterilized water (MDMA and Saline groups) or antibiotic cocktail mix water (A/M group) until the end of the experiment. Antibiotics consisting of Vancomycin (0.2 mg/mL), Bacitracin (0.5 mg/mL) and Neomycin (2 mg/mL) was chosen for broad spectrum coverage of bacteria with known lower systemic absorption as in previous experiments^5,43^. Fluid intake and body weight were monitored daily. Antibiotic cocktail was replaced every other day. Males and female rats respond differently to the hyperthermic effects of MDMA. We and others have published on these differences^44–47^. Given that we were wanting to specifically study the role of the microbiome in MDMA-induced hyperthermia, we used only male rats. Maintenance and research on animals were conducted in accordance with the eighth edition of the Guide for the Care and Use of Laboratory Animals, as adopted and promulgated by the National Institutes of Health. The Bowling Green State University Animal Care and Use Committee approved all the protocols and experimental procedures. All methods were carried out in compliance with relevant institutional, Federal and ARRIVE guidelines and regulations. At the completion of the study, animals were euthanized by carbon dioxide exposure.

### Drugs and chemicals

MDMA was obtained from Cayman Chemicals (Ann Arbor, MI, USA) as a hydrochloride salt. On the final day of the study, MDMA for injection was freshly prepared at a concentration of 20 mg/kg in 0.9% normal saline. Antibiotics Vancomycin, Bacitracin and Neomycin were purchased from Sigma Aldrich (St. Louis, MO, USA). BAs: Cholic Acid (CA), Chenodeoxycholic Acid (CDCA), Deoxycholic Acid (DCA) and deuterated Cholic acid (CA-D4) were also purchased from Cayman Chemical Company (Ann Arbor, MI).

### Research design and MDMA treatment

The study design for the experiment is outlined in Fig. 1. On the final day of the experiment (Day 6), fecal pellets were collected from rats in all three groups kept in sterilized bedding and cages one day prior. All treatments, blood draws and fecal samples were collected between 8 and 9 am on the day of treatment. Following that, rectal temperatures were measured as core body temperatures (Tc in °C) just prior to a single subcutaneous (sc) dose of MDMA (20 mg/kg) at time 0 min and recorded as baseline temperature. Until this point all three groups: Saline, MDMA and A/M represent groups before the MDMA/Saline challenge. Temperatures were further recorded at 30- and 60-min post MDMA/ Saline treatment for all groups. Physiotemp Thermalert TH-8 thermocouple (Physitemp Instruments, Clifton, NJ) attached to a RET-2 (rat) rectal probe was used for temperature measurement. Blood draws were made right after each temperature measurement at all time points. Serum from the blood draws (300 µL/time-point) and fecal pellets collected were stored at **– **20 °C or **− **80 °C until LC–MS/MS or DNA extraction.

### Bile acid and LC–MS/MS

CA, CDCA and DCA were dissolved in LC–MS grade methanol to a concentration of 1 mg/mL. Stock solutions of all 3 BA were then prepared at a concentration of 10,000 ng/mL followed by preparation of calibrators diluted to concentrations of: 5, 12.5, 25, 50, 250, 1250, 2500 and 5000 ng/mL. The CA-D4 was used as an internal standard for all BA prepared at a concentration of 1000 ng/mL in LC–MS grade methanol.

Mobile Phase A consisted of 771 mg of ammonium acetate (Fisher Chemical, Waltham, MA) in 900 mL of LC–MS grade water (Fisher Chemical, Waltham, MA) and 100 mL of LC–MS grade methanol (Fisher Chemical, Waltham, MA) with 1 mL of 25% ammonium hydroxide (Fisher Chemical, Waltham, MA) added. Mobile Phase B consisted of 771 mg of ammonium acetate in 1000 mL of methanol with 1 mL of 25% ammonium hydroxide added. Crash solution was prepared using Formic acid (LC–MS grade) and acetonitrile (LC–MS grade) purchased from Fisher Chemical (Waltham, MA). The compressed 5.0 ultra-high purity (UHP) grade argon gas tank used for the collision gas and compressed nitrogen gas tank for evaporation was obtained from Linde (Danbury, CT). A nitrogen generator to supply heating, drying, and nebulizing gases was from SouthTek (Wilmington, NC).

#### Serum purification methods

Serum was purified using protein precipitation with chilled crash solution (1% formic acid in acetonitrile). 300 µL of crash solution, 100 µL of serum and 60 µL of internal standard solution prepared in methanol were aliquoted to a 1.5 mL microcentrifuge tube and placed in an Eppendorf F1.5 ThermoMixer (Eppendorf, Enfield, CT) for 10 min at a speed of 1500 rpm. This was followed by centrifuging the crashed serum sample for 5 min at 6000×*g*. The supernatant was drawn off, transferred to a Hybrid SPE phospholipid filter (Supelco, Bellefonte, PA) and extracted by vacuum. Remaining acetonitrile in the extractant was then evaporated by a direct flow of nitrogen gas. The extractant were then reconstituted in 200 µL of Mobile Phase A and injected at an injection volume of 10 μL onto the liquid chromatography tandem mass spectrometer (LC–MS/MS).

#### Liquid chromatography tandem mass spectrometry methods

A triple-quadrupole LCMS-8050 CL from Shimadzu U.S.A (Canby, OR) was utilized for sample analysis with a gradient separation method. The flow rate was maintained at 0.50 mL/min at 50 °C with a total method run time of 6 min. The gradient began with 80% Mobile Phase A until 0.10 min and was then decreased to 53% Mobile Phase A. The gradient was then held at 53% Mobile Phase A until 2 min after which it decreased to 49% Mobile Phase A. The gradient was then decreased to 28% Mobile Phase A at 4.50 min and then switched to 0% Mobile Phase A at 4.51 min to wash the column. Additionally, the flow rate was changed to 0.8 mL/min for the duration of the 6-min run. The gradient was then reset to 100% Mobile Phase A at 5.01 min to re-equilibrate for the next sample. The stationary phase consists of a Raptor 50 mm × 2.1 mm, 2.7 µm, C18 column (Restek, Bellefonte, PA) for the separation of the analytes and a Raptor C18 guard column (Restek, Bellefonte, PA). Serum concentrations of the CA, CDCA and DCA were then quantified using LabSolutions Insight software (version 5.93). The calibration curve was prepared in SigMatrix Serum Diluent (Sigma-Aldrich, St. Louis, MO). All calibrators were subjected to the same extraction procedure as the samples.

### Microbial community analysis

16S rRNA gene sequencing was chosen as the method to characterize the gut bacterial communities of rats in the three treatment groups. DNA was extracted from ~ 250 mg offecal samples collected from a sterile setting from individual rats in each group using Dneasy Powersoil Pro Kit (Qiagen Inc., CA, USA) following the manufacturer’s instructions. Before sequencing, DNA was run in 0.8% agarose gel electrophoresis for quality check and concentration measured using NanoDrop Spectrophotometer (Thermo, MI, USA). For microbial analysis, all three groups: MDMA, A/M and Saline represent feces collected before the MDMA/Saline challenge.

Library preparation and sequencing of 16S rRNA gene libraries were performed by LC Sciences (Houston, TX, USA) using primers: Forward primer 338F (5ʹ-ACTCCTACGGGAGGCAGCAG-3ʹ) and Reverse primer 806R (5ʹ-GGACTACHVGGGTWTCTAAT-3ʹ) spanning V3-V4 region of ~ 450–460 bp amplicon length. Amplicon libraries were sequenced as 250- bp paired-end reads on an Illumina MiSeq platform. Raw sequence reads were trimmed to remove primers and low-quality ends, error-corrected and de-replicated using DADA2^48^ workflow 1.26.0 in R Studio (2022.07.1). This generated a total of 953,945 sequencing reads across the entire dataset after chimera removal. Taxonomies were assigned using Ribosomal Database Project (RDP) Classifier 16^49^. All libraries were rarefied to 49,400 reads prior to determination of alpha and beta diversities. Sequence from one animal (sample 5) in the antibiotic treated group was excluded from the analysis due to low sequence counts remaining after quality check. Principal coordinates analysis (PCoA) based on weighted Unifrac method and alpha diversity (observed ASVs and Shannon Index) measurements were performed using the Vegan package 2.6–4 in R.

To generate inferred metagenome (metabolic functional potential) information, the ASV table and reference sequences generated from DADA2 were used as input and run through PICRUSt2 pipeline^17^ in python (3.9). Both MetaCyc^50^ and KEGG databases^51^ were utilized to find the gene content in each of the samples. Normalization of gene counts in each sample was done by calculating the relative abundance of each gene per total gene count in the inferred metagenome for the sample.

### Statistical analysis

GraphPad InStat (v. 10.0) and GraphPad Prism (v. 10.0) were used for all statistical analyses, except otherwise stated for the microbiome analysis. Results are plotted as mean ± SEM for both rectal core body temperatures and BA data for all groups. One-way ANOVA with Student–Newman–Keuls or Dunnett’s (back to time zero) with post-hoc test was applied for comparing 3 groups and two-sample t-test for 2 groups. Significance used was at p < 0.05 a priori. For microbiome analysis, adonis test (PERMANOVA) was performed for compositional differences using weighted Unifrac distance method in R, and one way ANOVA non-parametric method based Kruskal–Wallis test with post-test (Dunn multiple comparison) for diversity measures and gene abundance analyses (for PICRUSt2).

## Data Availability

All data supporting the findings of this study are available within the paper.
